# Application of auditory mismatch negativity in tinnitus patients based on high-resolution electroencephalogram signals

**DOI:** 10.1515/tnsci-2022-0264

**Published:** 2022-12-08

**Authors:** Kunkun Wang, Xiaoling Lu, Shan Sun

**Affiliations:** ENT Institute and Otorhinolaryngology Department, Eye & ENT Hospital, State Key Laboratory of Medical Neurobiology and MOE Frontiers Center for Brain Science, Fudan University, Shanghai 200031, China; NHC Key Laboratory of Hearing Medicine, Fudan University, Shanghai 200031, China

**Keywords:** event-related potential, amplitude, auditory pathway, tinnitus

## Abstract

**Objective:**

The purpose of this study was to investigate the significance of mismatch negativity (MMN) by comparing high-resolution electroencephalogram signals from tinnitus patients and healthy controls.

**Methods:**

The study included eight subjects with chronic subjective idiopathic tinnitus and seven healthy controls. Participants with clinical speech (512–2,000 Hz) hearing thresholds less than 25 dB HL and with negative Hospital Anxiety and Depression Scale scores were included in the study. The E-Prime 2.0 software and a 256-electrode EGI Net Station system were used to evoke and record the MMN signal, and the amplitude and latency parameters of the MMN responses were compared between the two groups.

**Results:**

From 150 ms, there was a significant difference between the amplitude of standard stimulation and deviation stimulation, and the event-related potential amplitude under deviation stimulation in the tinnitus patient group was significantly different from that in the healthy group. The MMN amplitude of the FCz electrode was statistically significantly lower in the tinnitus patients compared to healthy controls.

**Conclusion:**

MMN has application value in the evaluation of abnormal electrical activity in the auditory pathway, and electroencephalograms are feasible for follow-up monitoring after acoustic therapy.

## 1 Introduction

Tinnitus describes the conscious perception of an auditory sensation without a corresponding external stimulus. Tinnitus is a common health problem worldwide, and it has an estimated prevalence of 8–30% in China [1]. Irritating tinnitus can make it difficult to fall asleep, and in serious cases, it can cause patients to become agitated, anxious, or even depressed, which will seriously affect daily work and quality of life. Currently, the neurophysiological origin of subjective tinnitus is unclear, but most researchers agree that tinnitus is linked to the central auditory pathways that play the predominant role in the increase in spontaneous activity [2]. Neural synchronization of the auditory cortex results from auditory deafferentation, changes in transient excitation potential, reorganization of the tonotopic map, and disordered regulation of the limbic system and auditory center, and these events form the pathophysiological basis of tinnitus [3,4]. Thus, research methods that can directly record changes in central nervous system electrical activity may become a powerful tool to study the mechanism of tinnitus.

Electroencephalography (EEG) records the information generated by the electrophysiological activities of neurons in the cerebral cortex. Extracellular currents travel along the brain and can be detected by EEG probes such that EEG fluctuations reflect local field potentials, which can be divided into spontaneous activity, evoked response, and induced response. Research based on the cortical self-generating activity and the event-related potential (ERP) is important for studying the central mechanism of tinnitus using EEG. ERPs have unique advantages in evaluating the auditory system and advanced brain function in tinnitus patients and have attracted more and more attention in both experimental and clinical research [5]. Over the past 20 years, studies have shown that spontaneous electrical activity in the cerebral cortex in tinnitus patients changes, including increases in the cortical slow wave rhythm (δ wave) and a decrease in α wave rhythm [6]. EEG has been used to study auditory ERPs, and it was found that the components of the auditory brainstem response and auditory steady-state response were altered in patients with tinnitus [7].

Mismatch negativity (MMN) is an important component of the auditory ERP, and MMN is triggered when a series of repeated sounds are suddenly interrupted by a deviant stimulus [8]. MMN is a negative component distributed in the forehead and center of the brain, and it is obtained using the oddball paradigm, which refers to the continuous alternating presentation of two or more different stimuli whose probabilities of occurring are significantly different. The standard stimulus is a stimulus with a higher probability (e.g., 70%), and the deviation stimulus is a stimulus with a lower probability (e.g., 30%). The ERPs of the standard stimulation and deviation stimulation are superimposed and averaged, and the ERP of the standard stimulation is subtracted from the ERP of the deviation stimulation to obtain the difference wave. The negative deflection over 100–250 ms after the occurrence of the deviant stimulation is the MMN [9]. Studies have shown that MMN is related to echo memory in the brain, and a pre-attention memory for about 30 s can be formed and stored when a simple auditory stimulus is first sensed [10,11,12]. Some studies suggest that abnormal function of the central NMDA receptor is the reason for the decrease in MMN seen in tinnitus patients [13,14,15], while others suggest that the decrease in MMN is related to inhibition of the reuptake of 5-HT from the presynaptic membrane [16,17,18]. The pathogenesis of tinnitus is not completely clear, and it may also be related to changes in endolymph potential in the cochlea. Changes in the neurotransmitters glutamate or serotonin may affect the symptoms or physiological processes of tinnitus [19,20,21,22,23], and the MMN of tinnitus patients and healthy people may be different and may be related to the function of neurotransmitter receptors [16]. In summary, MMN is a cognitive component of auditory ERP and is a specific reflection of the brain’s discrimination of auditory information, and it has the potential to be an objective indicator of tinnitus. This study mainly used MMN to observe the difference in EEG between tinnitus patients and healthy controls to explore the central auditory changes that occur in tinnitus and to explore the feasibility of using auditory MMN in tinnitus monitoring and evaluation.

## 2 Methods

### 2.1 Participants

**Figure 1:**
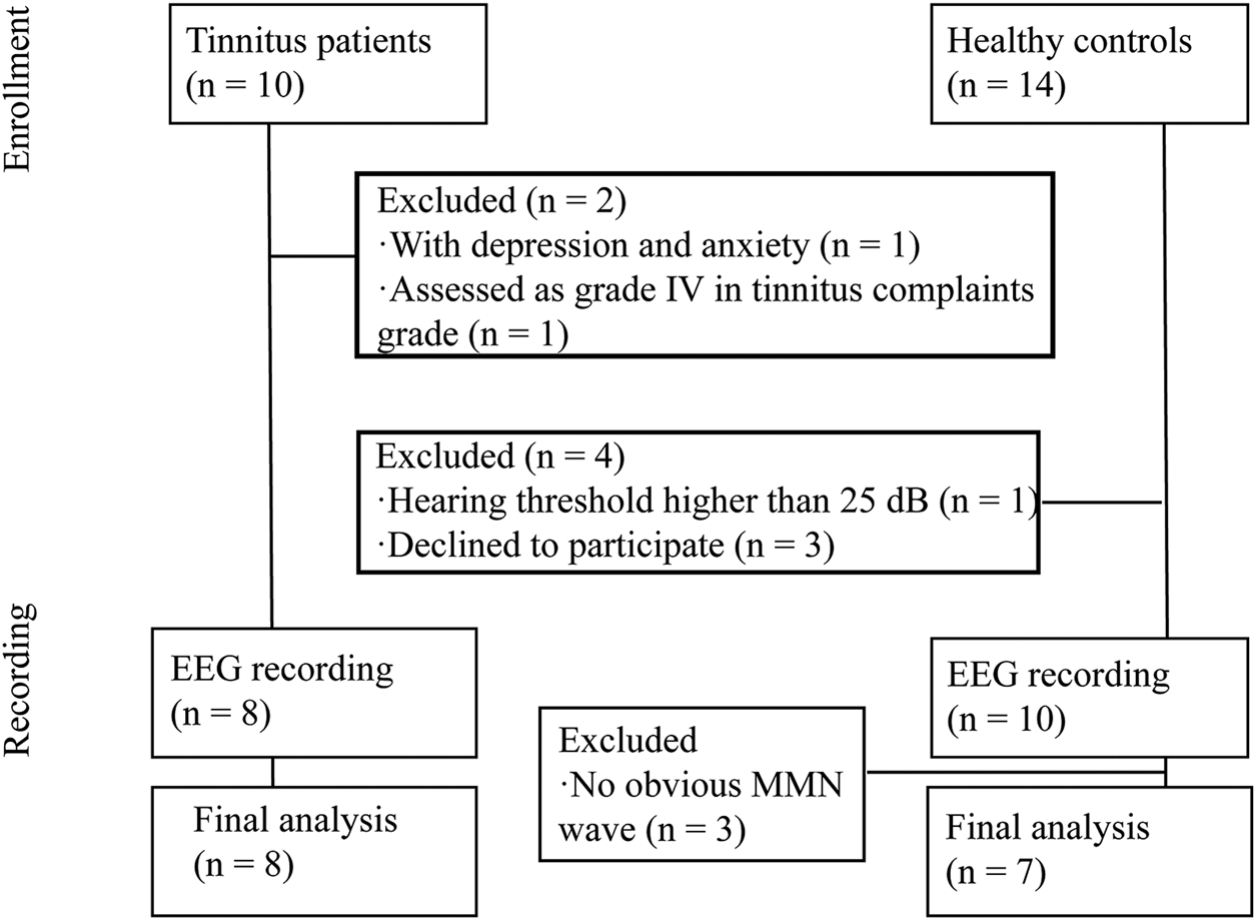
Flowchart of participants of the study. A total of 10 tinnitus patients and 14 healthy volunteers were enrolled in the study. One patient with tinnitus was excluded due to anxiety and depression, and another was excluded due to severe tinnitus rated as grade Ⅳ. One of the healthy volunteers was excluded because the hearing threshold exceeded 25 dB, and the other three chose not to participate in the study for personal reasons. Finally, eight patients and ten patients underwent EEG recording and three subjects in the control group failed to evoke MMN (amplitude < –0.35). Therefore, data for eight tinnitus patients and seven healthy controls were finally included in the analysis.

From April 2020 to December 2021, eight patients with tinnitus were included in this study. The inclusion criteria were (1) subjective chronic tinnitus and clinical speech hearing (512–2,000 Hz) thresholds less than 25 dB HL and (2) Hospital Anxiety and Depression Scale scores marked of fewer than seven points in the anxiety and depression parts. The exclusion criteria were (1) vascular pulsatile tinnitus; (2) otosclerosis, Meniere’s disease, acoustic neuroma, or other organic diseases of the auditory system; (3) being diagnosed with mental diseases such as anxiety or depression; (4) complications with severe systemic diseases; (5) being illiterate or unable to understand the content of the experiment or being unable to cooperate; and (6) being classified as grade Ⅳ according to Tinnitus Complaints Grade. At the same time, ten healthy volunteers without tinnitus were recruited under the same screening conditions (Figure 1). Finally, eight patients and ten healthy controls underwent EEG recording. Three subjects in the control group failed to evoke MMN (amplitude < –0.35 in any electrode). Therefore, data for eight tinnitus patients and seven healthy controls were included in the analysis.

The characteristics of the tinnitus patients were recorded in detail, including tinnitus side, tinnitus duration, tinnitus frequency, tinnitus volume, and the results of pure tone audiometry. A detailed questionnaire survey was conducted for each tinnitus patient, including tinnitus complaint grade [24], the Tinnitus Handicap Inventory, a visual analogue scale, and the Athens Insomnia Scale.


**Ethical approval:** The research related to human use has been complied with all the relevant national regulations, institutional policies and in accordance the tenets of the Helsinki Declaration, and has been approved by the Institutional Review Board of the Ethics Committee of the ENT Hospital of Fudan University (No. 2020010-1).
**Informed consent:** Informed consent has been obtained from all individuals included in this study.

### 2.2 EEG recordings and data analysis

The subjects were required to sit in a shielded room and to avoid shaking, chewing, and swallowing. During the experiment, the subjects watched a silent movie with subtitles and were told that there was no need to pay attention to any sounds in the environment or to touch any buttons. ERP data were recorded with an EGI 256-electrode system and were collected through E-Prime and net station platforms, and MMN wave EEG data were collected based on the oddball paradigm. The standard stimulus was 1 kHz pure tone, accounting for 85% of the auditory stimuli, and the deviation stimulus was 2 kHz pure tone, accounting for 15% of the auditory stimuli. The stimulus duration was 800 ms, and the interval between stimuli was 200 ms. Each group included a total of 1,000 instances of the standard stimulus and the deviation stimulus. The EEG activity that was generated synchronously with the transmission of standard stimulus and deviation stimulus was recorded. The resistances of all electrodes were kept below 10 kΩ.

EEG results were preprocessed in EEG lab, mainly including re-referencing based on the e257 electrode, low-frequency (0.5 Hz) denoising, interpolation of bad electrodes, and the removal of electro-oculogram and electromyogram waves based on the independent component analysis. ERP lab was then used to extract the ERP and to perform the averaging of the brain topographic map on the group level. The time point corresponding to the negative wave with the largest amplitude was calculated in the range of 100–250 ms using a self-made MATLAB script. The areas under the curve (AUCs) of MMN amplitude in the range of 100–250 ms were also taken into consideration.

Prism 8 was used for statistical analysis, and one-way analysis of variance (ANOVA) and independent samples’ *t*-tests were used for inter-group comparison between patients and healthy controls. One-way ANOVA was used for comparison between patient subgroups and healthy controls after dividing the groups into those younger than 40 years old and those older than 40 years, and Kruskal–Wallis tests were used for pairwise comparison. We choose the corresponding statistical methods according to the characteristics of their data and their normality and homogeneity of variance. The results were considered statistically significant if the *P*-value was less than 0.05.

## 3 Results

### 3.1 Descriptive analysis of the tinnitus and healthy groups

We divided the tinnitus patients into two subgroups according to their age so that the average age of the tinnitus patients younger than 40 years old did not differ significantly from the control group, whose mean age was 30.00 ± 4.28 years old (Table 1). The ratio of men to women was 4:3 in the control group and 4:4 in the tinnitus group. The pure tone audiometry result showed that the average hearing threshold for low frequency in the unilateral tinnitus group was lower than that in the bilateral tinnitus group (Table 2), and tinnitus patients with high frequency showed a higher hearing threshold. The severity of tinnitus showed no significant difference between the unilateral tinnitus and bilateral tinnitus groups, and the tinnitus characteristics were also relatively similar.

**Table 1:** Description of participants

	Control (*n* = 7)	Tinnitus (*n* = 8)			
Age (mean ± SD) (95% CI)	30.00 ± 4.28 (26.04, 33.96)	<40 years (*n* = 4)	*P* value	>40 years (*n* = 4)	*P* value
		34.25 ± 1.26 (32.25, 36.25)	ns vs control	57.00 ± 10.61 (40.11, 73.89)	<0.0001 vs control
Gender (M:F)	4:3	4:4			
Tinnitus duration (years) (95% CI)	/	2.10 ± 3.59 (−0.89, 5.11)			
Loudness matching of tinnitus (dB) (95% CI)	/	8.63 ± 5.93 (3.67, 13.58)			
Visual analogue scale (range 0–16) (95% CI)	/	6.13 ± 4.49 (2.38, 9.88)			
Tinnitus Handicap Inventory (range 0–100) (95% CI)	/	12.50 ± 23.54 (–7.18, 32.18)			
Athens Insomnia Scale (range 0–24) (95% CI)	/	3.00 ± 4.41 (−0.69, 6.69)			
Hospital Anxiety and Depression Scale (range 0–42) (95% CI)	/	3.50 ± 5.01 (−0.69, 7.69)			

**Table 2:** Clinical characteristics of tinnitus patients

	Unilateral (*n* = 4)	Bilateral (*n* = 4)
Pure tone audiometry thresholds (dB)		
125 Hz (L; R)	18.75; 22.5	16.25; 16.25
250 Hz (L; R)	17.5; 21.25	16.25; 13.72
500 Hz (L; R)	20; 26.25	17.5; 15
1,000 Hz (L; R)	17.5; 27.5	17.5; 16.25
2,000 Hz (L; R)	21.25; 25	23.75; 25
4,000 Hz (L; R)	27.5; 36.25	53.75; 51.25
8,000 Hz (L; R)	23.75; 31.25	52.5; 53.75
Pitch matching of tinnitus (Hz)	4,000 (2); 8,000 (2)	4,000 (2); 8,000 (2)
Tinnitus complaints grade (I–V)	II (2); III (2)	II (2); III (2)

### 3.2 Comparison of EEG between the tinnitus and healthy groups

**Figure 2:**
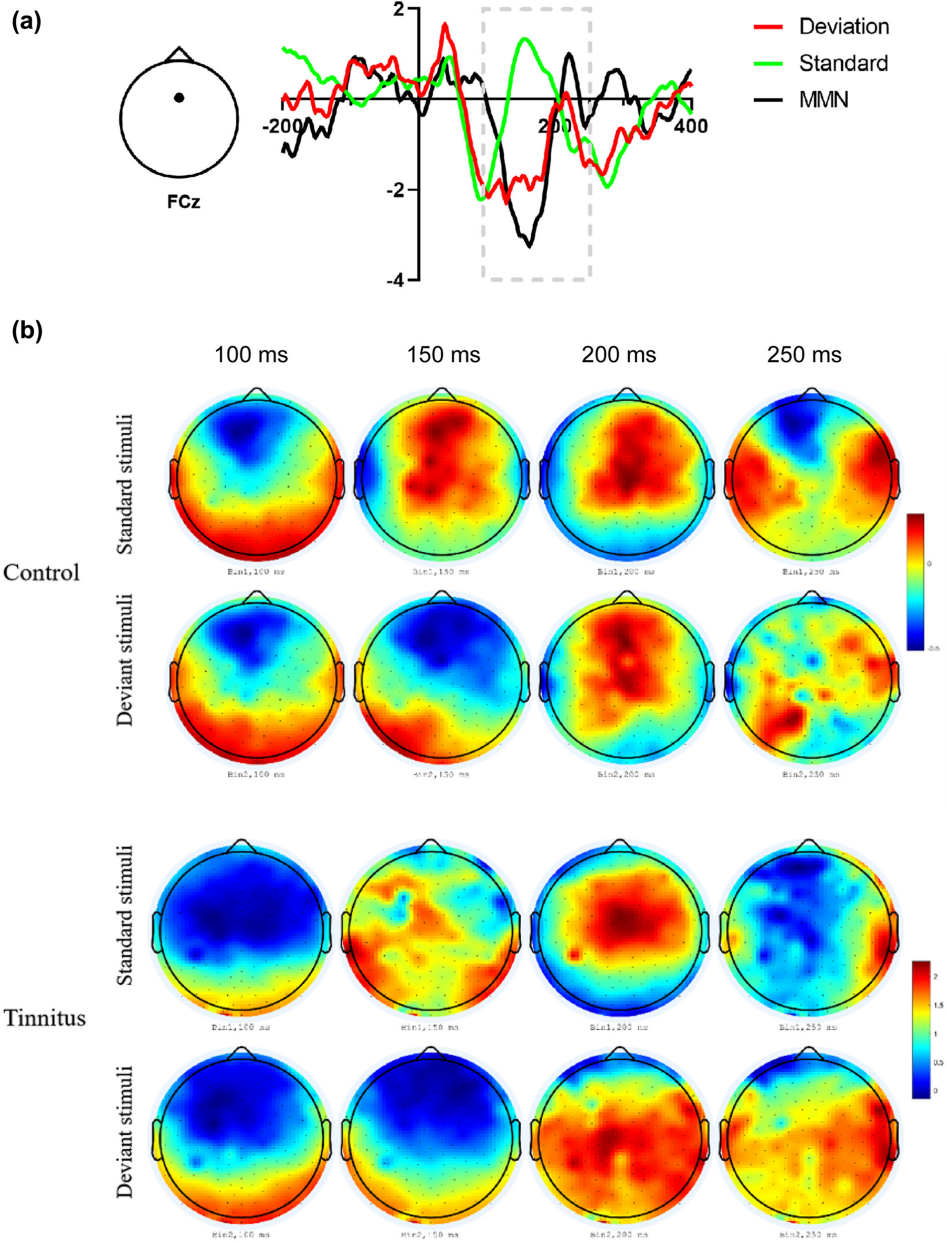
Typical MMN components that are extracted from the FCz electrode. (a) The image on the left is the vertical view of the head. The triangle represents the nose pointing upward, and the dots represent the positions of the FCz electrodes where the MMN signal was collected. The abscissa is the latency (ms), the ordinate is the amplitude, the red line represents the deviation stimulus, and the green line represents the standard stimulus. Subtracting the amplitude of the standard stimulus from the amplitude of the deviation stimulus provides the MMN, as represented by the black line. (b) ERP topographic maps in the brains of the control and tinnitus groups under standard stimulation and deviation stimulation at 100, 150, 200, and 250 ms.

**Figure 3:**
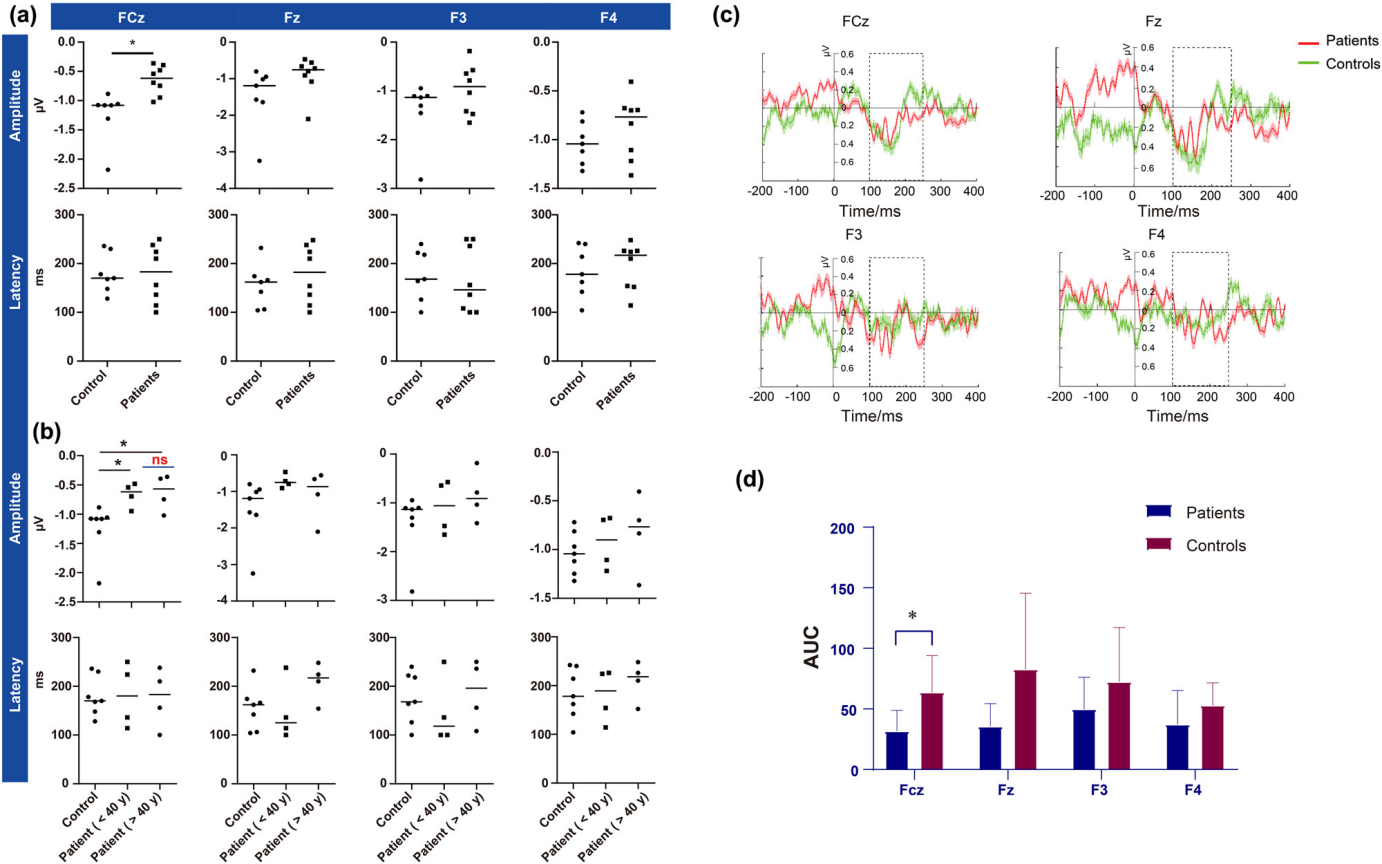
Amplitudes and latencies of the FCz, Fz, F3, and F4 electrodes in the control group and the tinnitus group. (a) There was a significant difference between the groups in the FCz amplitude. There was no significant difference between the two groups for the other electrodes (*t*-test, *p < 0.05). (b) The FCz amplitude of tinnitus patients both younger and older than 40 years old was lower than that of healthy controls. There was no significant difference between subgroups of patients, and there was no significant difference among the three groups for the other electrodes (one-way ANOVA, *p < 0.05). (c) The ERP diagram of tinnitus patients and the control group with FCz, Fz, F3, and F4 leads. Red represents the tinnitus patient group, and green represents the control group. The dotted line shows the waveform within 100–250 ms, which is the area selected for calculating MMN in this study. (d) The AUC of MMN amplitudes of the FCz, Fz, F3, and F4 electrodes in the control group and the tinnitus group. Unpaired *t*-test showed that the amplitude of MMN in the FCz electrode of patients was lower than that of the control group, which was consistent with the statistical results using the maximum value to calculate MMN (*t*-test, *p < 0.05).

MMN is mainly concentrated in the auditory cortex and its adjacent cortex and frontal lobe. The induction of the Fz electrode MMN was obvious in the midline. Therefore, this study mainly collected the MMN components of the Fz, FCz, and nearby electrodes for analysis. As shown in Figure 2a, the typical MMN component waveform was obtained by extracting the potential amplitude under standard stimulation and deviation stimulation and then subtracting the former from the latter. ERP analysis was carried out for latencies of 100, 150, 200, and 250 ms (Figure 2b). There was no obvious MMN amplitude component in the control group or the tinnitus group at 100 ms, while from 150 ms, there was a significant difference between the amplitudes of the standard stimulation and the deviation stimulation. The MMN amplitudes and latencies of the Fz, FCz, F3, and F4 electrodes of the control group and tinnitus patient group were recorded and compared, and the MMN amplitude of the FCz electrode was statistically significantly lower in tinnitus patients compared to healthy controls in terms of the maximum value or the AUC of MMN amplitude (Tables 3 and 4; Figure 3).

**Table 3:** Amplitude and latency of tinnitus patients and healthy controls

		Patients (*n* = 8) mean ± SEM	Control (*n* = 7) mean ± SEM	*t*-value	*P*-value	95% CI
F3	Amplitude	−0.97 ± 0.18	−1.41 ± 0.24	1.45	0.17	(−0.22, 1.10)
	Latency	167.00 ± 23.93	176.90 ± 19.79	0.32	0.76	(−77.04, 57.33)
F4	Amplitude	−0.88 ± 0.11	−1.03 ± 0.08	1.11	0.29	(−0.15, 0.47)
	Latency	194.30 ± 16.84	183.10 ± 19.57	0.43	0.67	(−44.96, 67.17)
FCz	Amplitude	−0.65 ± 0.09	−1.24 ± 0.16	3.18	0.011*	(0.17, 1.01)
	Latency	178.50 ± 20.85	179.70 ± 15.13	0.05	0.96	(−57.18, 54.75)
Fz	Amplitude	−0.91 ± 0.18	−1.49 ± 0.32	1.58	0.15	(−0.24, 1.39)
	Latency	178.00 ± 20.77	155.10 ± 16.64	0.86	0.41	(−34.76, 80.47)

*t*-test, *p < 0.05.

**Table 4:** The area under the curve of MMN amplitude of patients and controls

	Patients (*n* = 8) mean ± SEM	Control (*n* = 7) mean ± SEM	*t*-value	*P*-value	95% CI
F3	49.95 ± 9.22	72.39 ± 16.98	1.20	0.25	(−17.84, 62.73)
F4	37.20 ± 9.91	52.89 ± 7.05	1.26	0.23	(−11.32, 42.69)
FCz	31.76 ± 6.09	63.69 ± 11.51	2.54	0.024*	(4.82, 59.03)
Fz	35.59 ± 6.65	82.69 ± 23.76	2.03	0.064	(−3.70, 97.28)

*t*-test, *p < 0.05.

Furthermore, we performed the comparison between the control group and tinnitus subgroups, and the results showed that the FCz amplitude was different in the control group compared to both patients younger than 40 years old and patients older than 40 years old. And similar situation was observed between young patients and controls in terms of Fz amplitude (Table 5). The results above indicate that the MMN amplitude of the FCz and Fz electrodes in patients with tinnitus is lower than that in healthy people, despite the different age groups.

**Table 5:** Comparison of the amplitude and latency between subgroups of tinnitus patients and healthy controls

		Patients (*n* = 8) mean ± SEM		*P*-value		
		<40 years (*n* = 4)	>40 years (*n* = 4)	ANOVA	<40 years vs control	>40 years vs control
F3	Amplitude	−0.93 ± 0.21	−1.01 ± 0.33	0.49	0.44	0.98
	Latency	146.5 ± 35.53	187.5 ± 33.63	0.59	0.88	>0.99
F4	Amplitude	−0.99 ± 0.18	−0.76 ± 0.15	0.40	>0.99	0.32
	Latency	193.5 ± 26.74	195.0 ± 24.66	0.90	>0.99	>0.99
FCz	Amplitude	−0.68 ± 0.12	−0.61 ± 0.14	0.0022**	0.042*	0.016*
	Latency	186.0 ± 31.12	171.0 ± 31.98	0.93	>0.99	>0.99
Fz	Amplitude	−0.68 ± 0.10	−1.14 ± 0.33	0.041*	0.030*	0.63
	Latency	149.5 ± 33.66	206.5 ± 18.41	0.28	>0.99	0.37

One-way ANOVA, *p < 0.05, **p < 0.01.

## 4 Discussion

Most of the tinnitus patients are accompanied by hearing loss, but the subjects recruited to this study were excluded if they had primary otological diseases or other serious systemic diseases. In addition, to avoid the impact of potential emotional abnormalities on the EEG data, we specifically excluded patients with grade Ⅳ tinnitus according to the Tinnitus Complaints Grade. Therefore, the data quality of this study is relatively good. In addition, we used a high-resolution EGI 256-electrode system to obtain more reliable data.

Previous studies have shown that when the difference between deviant stimuli and standard stimuli increases, the amplitude of the MMN, which is caused by the increase in auditory adaptation, increases accordingly [25]. At the same time, MMN may reflect the pre-attentive stages of information processing and sensory analysis of auditory input and the process of encoding this auditory input into memory traces [26]. In studies of other auditory diseases, it was found that the amplitude of MMN is related to the severity of aphasia and to the severity of auditory cortex damage [27]. When the amplitude of MMN increases, language processing improves, which means that the increase in MMN amplitude is related to the improved function of the auditory cortex [28], and another study reached the same conclusion [29]. In our study, the amplitude of MMN in tinnitus patients was significantly lower than that in the healthy controls, which verified that the pathophysiology of tinnitus originates in the central nervous system.

We used multiple electrodes to record EEG data, and we collected the electrode data near the midline of the frontal lobe because the evoked potential is the most obvious here. Although the function of the central frontal lobe is mainly related to autokinesis, the frontal lobe itself is also related to the auditory system. It is known that the central auditory cortex plays an important role in the development and maintenance of tinnitus, but brain regions other than the auditory system such as the prefrontal cortex and the limbic system also have an impact on tinnitus [30]. Some studies have applied magnetic resonance imaging to explore the difference in the volume and density of different brain regions between patients and healthy controls, and they have concluded that the gray matter is increased in the auditory thalamus [31] and anterior cingulate and frontal cortex [32] in tinnitus patients, while gray matter is decreased in the ventromedial prefrontal cortex [33,34] and the superior frontal cortex [35,36]. The studies above demonstrated the feasibility of analyzing electrode data near the midline of the frontal lobe.

As a general indicator of brain plasticity, MMN can be used to study the development and decline of memory traces following any form of experimental stimulation. Previous studies also showed that MMN has application value in the evaluation of abnormal electrical activity in the auditory pathway [37,38]. Pekkonen et al. [39] found that in the case of short stimulation (0.5 s), the MMN amplitude of young and elderly subjects was similar, suggesting that the auditory memory trace discrimination that is prompted by MMN is not affected by age. In our preliminary analysis, the amplitude of the FCz electrode in tinnitus patients was different from healthy people. To eliminate the limitations of age mismatch, we divided the patients into those younger than 40 years and those older than 40 years, and the results showed that the amplitude of the FCz electrode in both the younger tinnitus patients and the older patients was lower than that in healthy controls. The age of the overall patients did not match the age of the control group. There may be changes in the MMN due to the decline in the automatic processing of brain information or to the ability to trace auditory memory due to the increase in age. When the patients were grouped by age and then compared, there might be a bias in the results due to the small sample size. However, in general, our statistical analysis shows a decreasing trend of MMN in tinnitus patients compared with healthy controls, and we hope that this can supplement the database of existing studies and provide enlightenment related to research methods or results for future studies.

Some researchers have explored changes in MMN in patients with tinnitus, and their results have shown that the MMN amplitude in patients with tinnitus is lower than that in healthy people, which is consistent with our findings [38,40,41]. Based on these findings, MMN has been used to study the auditory sensory changes related to residual auditory inhibition in patients with tinnitus [42]. However, the clinical application of MMN in tinnitus patients is still rare. Our results show that the amplitude of MMN in tinnitus patients is significantly lower than that in healthy controls, thus suggesting that the MMN value can be used in the evaluation of tinnitus patients.

## 5 Conclusion

Differences in the amplitude of MMN, which is an EEG component of the classical auditory pathway, between healthy controls and tinnitus patients were significant in the central frontal region, suggesting that the amplitude of MMN may play a certain role in evaluating tinnitus, which can pave the way for further research with larger sample size and better design, and may have clinical potential for the diagnosis or evaluation of tinnitus patients, which is worth further study.
